# A Chlorhexidine- Agar Plate Culture Medium Protocol to Complement Standard Broth Culture of *Mycobacterium tuberculosis*

**DOI:** 10.3389/fmicb.2016.00030

**Published:** 2016-01-25

**Authors:** Shady Asmar, Sonia Chatellier, Caroline Mirande, Alex van Belkum, Isabelle Canard, Didier Raoult, Michel Drancourt

**Affiliations:** ^1^URMITE, UMR CNRS 7278, IRD 198, INSERM 1095, Aix Marseille UniversitéMarseille, France; ^2^Innovation Unit, BioMérieux SALa Balme Les Grottes, France; ^3^R&D Microbiology, BioMérieux SALa Balme Les Grottes, France

**Keywords:** *Mycobacterium tuberculosis*, diagnosis, decontamination, chlorhexidine, MOD9, Bactec 960 MGIT, N-acetyl-cysteine-sodium chloride

## Abstract

The culture of *Mycobacterium tuberculosis* using parallel inoculation of a solid culture medium and a liquid broth provides the gold standard for the diagnosis of tuberculosis. Here, we evaluated a chlorhexidine decontamination-MOD9 solid medium protocol versus the standard NALC-NaOH-Bactec 960 MGIT protocol for the diagnosis of pulmonary tuberculosis by culture. Three-hundred clinical specimens comprising 193 sputa, 30 bronchial aspirates, 10 broncho-alveolar lavages, 47 stools, and 20 urines were prospectively submitted for the routine diagnosis of tuberculosis. The contamination rates were 5/300 (1.7%) using the MOD9 protocol and 17/300 (5.7%) with the Bactec protocol, respectively (*P* < 0.05, Fisher exact test). Of a total of 50 Mycobacterium isolates (48 M. tuberculosis and two *Mycobacterium abscessus*) were cultured. Out of these 50, 48 (96%) isolates were found using the MOD9 protocol versus 35 (70%) when using the Bactec protocol (*P* < 0.05, Fisher exact test). The time to positivity was 10.1 ± 3.9 days versus 14.7 ± 7.3 days, respectively, (*P* < 0.05, Student’s *t*-test). These data confirmed the usefulness of parallel inoculation of a solid culture medium with broth for the recovery of *M. tuberculosis* in agreement with current recommendations. More specifically, chlorhexidine decontamination and inoculation of the MOD9 solid medium could be proposed to complement the standard Bactec 960 MGIT broth protocol.

## Introduction

Between 1990 and 2013, the mortality rate of tuberculosis fell 45% due to global efforts by the world health organization (WHO) impending the 2015’s millennium development goals (World Health Organization [WHO], 2014). Still, pulmonary tuberculosis claims 1.5 million human lives every year, of which 95% occur in low and middle income countries (World Health Organization [WHO], 2014). Culture of *Mycobacterium tuberculosis* and related mycobacteria remains the gold standard for the laboratory diagnosis (Ghodbane et al., 2014). Recently, we showed that MOD9, a newly developed solid culture medium, was superior to the standard Löwenstein–Jensen medium in terms of increased sensitivity and reduced time-to-positive culture of *M. tuberculosis* (Asmar et al., 2015). Moreover, this medium is compatible with chlorhexidine decontamination, which has been favorably evaluated for the diagnosis of tuberculosis (Asmar and Drancourt, 2015) and non-tuberculosis mycobacteria (NTM) (Ferroni et al., 2006). However, combining chlorexhidine decontamination with MOD9 medium has not been compared to the standard NALC-NaOH/Bactec MGIT protocol routinely used and we are reporting on such evaluation.

## Materials and Methods

### Specimens

This work has received the agreement of the Institut Fédératif de Recherches 48 Ethics Committee on February 19, 2007. A total of 300 clinical specimens prospectively analyzed in this study, including 250 clinical specimens of a previous report comparing MOD9 and Löwenstein–Jensen culture (Asmar et al., 2015) included 233 (77.6%) respiratory tract specimens [193 sputa (82.8%), 30 bronchial aspirates (12.9%) and 10 broncho-alveolar lavages (4.3%)] and 67 (22.4%) non-respiratory tract specimens [47 stools (70.1%) and 20 urines (29.9%)] were received from 156 patients clinically suspected with tuberculosis. Eighty-three patients (53.2%) gave one specimen, 41 patients (26.3%) two specimens, 17 patients (10.9%) three specimens, five patients (3.2%) four specimens, four patients (2.6%) five specimens, three patients (1.9%) six specimens, two patients (1.3%) eight specimens and one patient (0.6%) gave 10 specimens. Ziehl–Neelsen smears were prepared for all respiratory tract specimens and examined under light microscopy at × 1,000 magnification.

### Specimens Processing

In the standard broth protocol A, respiratory tract and stools specimens decontaminated using the reference NALC-NaOH method (Becton Dickinson, Le Pont-de-Claix, France) (Ghodbane et al., 2014) were inoculated into a MGIT tube (Becton Dickinson) supplemented with 500 μL of PANTA antibiotic cocktail (Becton Dickinson). Tubes were incubated in the automated Bactec 960 system V5.01A (Becton Dickinson). In parallel, leftovers of the respiratory tract specimens (100 μL – 1 mL) were decontaminated using 0.7%-chlorhexidine (Asmar and Drancourt, 2015) and inoculated on MOD9 culture medium (Asmar et al., 2015) (protocol B). As for stool specimens, 10 μL were recovered using a sterile loop and suspended in two mL of sterile PBS, then an equal volume of NALC-NaOH (for BACTEC inoculation) or a triple volume of 0.7%-chlorhexidine (for MOD9 inoculation) were added and the rest of the procedure was followed as described above. MOD9 plates were inspected by the naked-eye every 24 h for the presence of colonies for 4 weeks.

All isolates were identified by Ziehl–Neelsen staining and real-time qPCR as previously described (Asmar et al., 2015).

### Statistical Analyses

The student’s *t-*test was used to compare the growth detection times, and Fisher exact test was used to assess the significance of differences in the mycobacterial isolation rates and contamination rates. The difference was considered significant when *P* < 0.05.

## Results and Discussion

The contamination rate of 17/300 (5.7%) including nine stools, seven sputa and one bronchial aspirate in routine protocol A, was significantly higher than the 5/300 (1.7%) contamination rate in challenging protocol B, including four stools and one sputum (*P* < 0.05, Fisher’s exact text). The five specimens that grew contaminants with protocol A also grew contaminants in protocol B. This observation is confirmatory of what we previously reported (Asmar and Drancourt, 2015).

**FIGURE 1:**
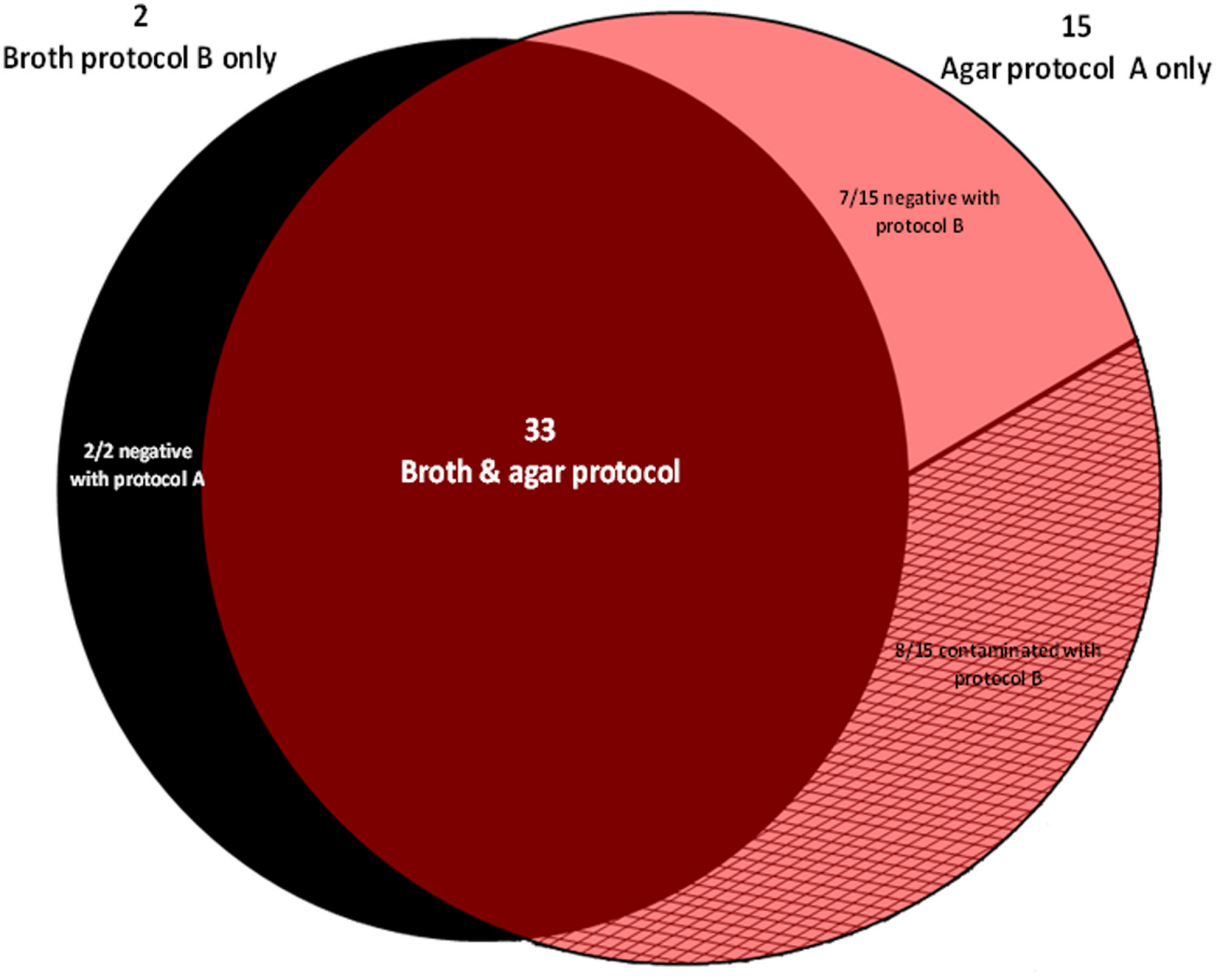
**Distribution of 50 *Mycobacterium* isolates yielded by the broth protocol B (NALC-NaOH/Bactec 960 MGIT) and the agar protocol A (chlorhexidine-0.7%/MOD9) out of 300 inoculated specimens**.

A total of 50/300 specimens (16.7%) from 19 patients (12.2%) grew mycobacteria, including 48/50 isolates (96%) from 17 patients identified as *M. tuberculosis*, while the two remaining isolates made in two additional patients were identified as *Mycobacterium abscessus*. These 300 specimens included 22 smear-positive specimens which grew *M. tuberculosis* in 21 specimens and *M. abscessus* in one specimen; and 278 smear-negative specimens, which grew 27 *M. tuberculosis* and one *M. abscessus* (*P* < 0.05, Fisher’s exact text). Thirty-three out the 50 positive specimens (66%) isolated from 14 different patients were positive using both protocols A and B. Bactec 960 MGIT yielded 35 *Mycobacterium* isolates (70%), identified as 34 *M. tuberculosis* [34/48 (70.8%)] (in 26 sputa, four stools and four bronchial aspirations) and one *M. abscessus* (in one sputum). MOD9 yielded 48 *Mycobacterium* isolates (96%), identified as 46 *M. tuberculosis* [46/48 (95.8%)] (in 31 sputa, 11 stools and four bronchial aspiration specimens) and two *M. abscessus* (in two sputum specimens) (*P* < 0.05 Fisher’s exact test). Two *M. tuberculosis* isolates (one sputum and one stool specimen) were recovered only by Bactec 960 MGIT, while 14 *M. tuberculosis* isolates (in six sputa and eight stool specimens) and one *M. abscessus* isolated from a sputum specimen grew only on MOD9 medium. Eight (53.3%) isolates (two sputa and six stools) were lost in MGIT due to contamination (**Figure 1**). We estimated that the MOD9 medium itself could account for 46.7% of strains isolated in excess in protocol B.

The mean time to detection of *M. tuberculosis* in Bactec 960 MGIT was 14.7 ± 7.3 (4–32) days [Smear positive specimens (19) 10.3 ± 4.9 (4–21) days; smear-negative specimens (15) 20.4 ± 5.7 (7–32) days (*P* < 0.05 Student’s *t*-test)] versus 10.1 ± 3.9 (4–18) days using protocol B [smear positive specimens (21) 6.8 ± 1.6 (4–9) days; smear-negative specimens (25) 12.8 ± 3.1 (8–18) days (*P* < 0.05 Student’s *t*-test)] (*P* < 0.05 Student’s *t*-test) as shown in **Table 1**. Furthermore, the mean time to detection of *M. tuberculosis* grown in common on both MOD9 and Bactec 960 MGIT (*n* = 32) was significantly lower for protocol B [8.8 ± 3.4 (4–18) days] than in broth protocol A [13.9 ± 6.5 (4–25) days] (*P* < 0.05, Student’s *t*-test) as shown in **Table 1**.

**Table 1:** Comparison of chlorhexidine-0.7%/MOD9 and NALC-NaOH/Bactec 960 MGIT protocols for the culture of mycobacteria in 300 clinical specimens.

	NALC-NaOH/Bactec 960 MGIT		0.7%-chlorhexidine/MOD9	
**Sensitivity (*Mycobacterium tuberculosis*)**	70.8% (34/48)		95.8% **^∗^** (46/48)	

**Contamination rate**	5.7% (*n* = 17)		1.7% **^∗^** (*n* = 5)	

Mean time to detection *M. tuberculosis* (days)	14.7 ± 7.3 (4–32) (*n* = 34)		10.1 ± 3.9 (4–18) **^∗^** (*n* = 46)	
	ZN+	ZN-	ZN+	ZN-
	10.3 ± 5 (4–21) (*n* = 19)	20.4 ± 5.7 (7–32) (*n* = 15)	6.8 ± 1.6^∗^ (4–9) (*n* = 21)	12.8 ± 3.2^∗^ (8–18) (*n* = 27)

Mean time to detection of *M. tuberculosis* growth in both MGIT and on MOD9 (days)	13.9 ± 6.5 (4–25) (*n* = 32)		8.7 ± 3.3 (5–18) ^∗^ (*n* = 32)	
	ZN+	ZN-	ZN+	ZN-
	10.3 ± 4.9 (4–21) (*n* = 19)	19.2 ± 4.8 (7–25) (*n* = 13)	6.8 ± 1.7^∗^ (4–9) (*n* = 19)	11.7 ± 3.4^∗^ (8–18) (*n* = 13)

*^∗^denotes *P* < 0.05 Fisher’s exact test; ZN denotes Ziehl–Neelson Staining.*

Altogether, a total of 282 specimens were free of any contaminant. These specimens yielded a total of 42 isolates including 40 *M. tuberculosis* and two *M. abscessus* isolates. Considering these 282 specimens, the sensitivity of agar and broth protocol for culturing *M. tuberculosis* was of 95 and 85%, respectively. The mean time to detect *M. tuberculosis* growth was significantly lower for protocol B (9.3 ± 3.9 days) than for protocol A (14.5 ± 7.3 days) (*P* < 0.05, Student’s *t-*test).

The data here reported confirm the usefulness of the chlorhexidine/MOD9 protocol as an alternative to or as a complement of the MGIT protocol as chlorexhidine is not compatible with MGIT broth. The chlorexidine / MOD9 protocol could be used to complement any broth-based culture by a solid medium-based culture for the diagnosis of tuberculosis and non-tuberculosis mycobacterioses, in line with current recommendations for the culture-based diagnosis of these infections (Nolte and Metchock, 1999).

## Author Contributions

All authors listed, have made substantial, direct and intellectual contribution to the work, and approved it for publication.

## Conflict of Interest Statement

The authors are co-inventors of a patent related to the MOD-9 medium here reported.
